# Pharmacokinetics of dexmedetomidine in anaesthetized horses following repeated subcutaneous administration and intravenous constant rate infusion

**DOI:** 10.1186/s12917-023-03831-w

**Published:** 2023-12-09

**Authors:** Federica Di Cesare, Vanessa Rabbogliatti, Susanna Draghi, Martina Amari, Federica Alessandra Brioschi, Roberto Villa, Giuliano Ravasio, Petra Cagnardi

**Affiliations:** Department of Veterinary Medicine and Animal Sciences, Università degli Studi di Milano, Milan, Italy

**Keywords:** Balanced anaesthesia, Constant rate infusion, Dexmedetomidine, Equine patient, Liquid Chromatography Mass Spectrometry, Pharmacokinetics, Subcutaneous

## Abstract

**Background:**

The inclusion of dexmedetomidine (DEX) within a balanced general anaesthesia protocol is effective in improving the clinical outcome and recovery quality of anaesthesia in horses. This study aimed to determine the pharmacokinetic profile of DEX following repeated subcutaneous (SC) administration at 2 µg/kg every 60 min till the end of the procedure in comparison to intravenous constant rate infusion (CRI) at 1 µg/kg/h in anaesthetized horses undergoing diagnostic procedures up to the end of the diagnostic procedure.

**Results:**

In the CRI and SC groups DEX maximum concentrations (C_max_) were 0.83 ± 0.27 ng/mL and 1.14 ± 0.71 ng/mL, respectively, reached at a time (T_max_) of 57.0 ± 13.4 min and 105.5 ± 29.9 min. Mean residence time to the last measurable concentration (MRT_last_) was 11.7 ± 6.2 and 55.8 ± 19.7 min for the CRI group and SC groups, respectively. The apparent elimination half-life was 18.0 ± 10.0 min in the CRI group and 94.8 ± 69.8 min for the SC group, whereas the area under the curve (AUC_0-last_) resulted 67.7 ± 29.3 and 83.2 ± 60.5 min*ng/mL for CRI and SC group, respectively. Clearance was 16.26 ± 8.07 mL/min/kg for the CRI group. No signs of adverse effects were recorded in both groups.

**Conclusions:**

The pharmacokinetic profile of DEX following repeated SC administration in anaesthetized horses was comparable to intravenous CRI administration during the intranaesthetic period and beneficial during the recovery phase from general anaesthesia. The SC route could be considered as an alternative to CRI for improving the recovery quality of equine patients undergoing general anaesthesia.

**Supplementary Information:**

The online version contains supplementary material available at 10.1186/s12917-023-03831-w.

## Introduction

General anaesthesia in equine patients carries a higher risk compared to humans and small animals [1, 2]. To mitigate this risk, a balanced anaesthetic protocol involving the use of inhalant or injectable anaesthetics in combination with short-acting adjuvants for maintaining good intraoperative cardiopulmonary function is recommended [3, 4].

Alpha-2-adrenergic receptor agonists represent a cornerstone within equine balanced sedative and anaesthetic protocols [5–7]. In fact, in horses, when administered intravenously as a bolus or as a constant rate infusion (CRI), these drugs contribute to provide sedation and analgesia also reducing, during general anaesthesia the inhalant anaesthetic requirements [5, 8, 9]. Additionally, in this species, the use of alpha-2-adrenergic receptor agonists has been also reported to improve the quality of recovery from general anaesthesia [8, 10, 11].

Dexmedetomidine (DEX) is the most selective and potent alpha-2-adrenergic receptor agonist that has been investigated in horses as an ideal adjuvant agent for sedation and balanced anaesthesia due to its beneficial pharmacological profile [6, 12–14].

In equine practice, the most common DEX administration route employed for either sedation or general anaesthesia is the intravenous one, delivered as a bolus or as a CRI, with several studies that investigated the pharmacokinetics and the pharmacodynamics of this drug for clinical purposes [10, 12, 13, 15–18]. Conversely, information regarding other types of DEX administration routes in horses is limited. Only two studies explored alternative delivery routes in this species, in the study by Shane et al., (2021) [12], DEX pharmacokinetic profile and pharmacodynamic properties were determined following CRI and repeated intramuscular injections administered for standing sedation. The other work, recently published by the authors of the present investigation, evaluated DEX clinical effects within the intranaesthetic period and the recovery phase when administered as CRI or following repeated subcutaneous (SC) injections [19]. On the other hand, DEX pharmacokinetic profile following SC administration has never been described in veterinary medicine.

In veterinary medicine, the SC route for anaesthetic and analgesic adjuvants administration is mainly employed in feline and rabbit patients due to its practical convenience [20–22]. In other species, such as canine and equine, the use of the SC route is still controversial due to the pharmacokinetic behaviour mainly characterized by high intersubject variability which impredictably may result in clinical ineffectiveness [23–25].

In humans, the SC administration of DEX demonstrated to be adequately absorbed inducing a minimal hemodynamic impact compared to the IV administration [26, 27]. The clinical use of DEX repeated SC administration in anaesthetized horses demonstrated positive results, with a better recovery quality from general anaesthesia in comparison with CRI, highlighted by the reduced attempts to stand and lower ataxia [19]. It might be hypothesized that the CRI and the repeated SC administration would display a comparable pharmacokinetic profile during the intranaesthetic period and that SC would present a beneficial pharmacokinetic behaviour during the recovery phase. Hence, the present study aimed to determine and compare the pharmacokinetic profile of DEX following intravenous CRI and repeated SC administration in horses within a balanced anaesthetic protocol for magnetic resonance examination.

## Materials and methods

### Animals and study design

Within the larger parallel group used in the DEX clinical evaluation [19], twenty adult, client-owned, non-food producing horses (aged from 6 to 18 years and weighing from 388 to 620 kg) were randomly (www.randomizer.org) selected to undergo the DEX pharmacokinetic study after either IV CRI (*n* = 10; CRI group) or repeated SC administration (*n* = 10; SC group) (Table 1).

The inclusion criterium was an American Society of Anesthesiologists (ASA) physical status I or II based on clinical examination and blood work, moreover, horses that received any medications in the 30 days before the study were excluded. Animals were presented at the Veterinary Teaching Hospital of the University of Milan (Lodi, Italy) to undergo magnetic resonance examination under general anaesthesia.

The study protocol was approved by the Institutional Ethical Committee for Animal Care at the University of Milan (OPBA_17_2020), and all horses were enrolled after obtaining written consent from the owners. All procedures were carried out in accordance with the relevant guidelines and regulations and the study was carried out in compliance with the CONSORT and ARRIVE 2.0 guidelines. All horses were discharged from the Veterinary Teaching Hospital after a 24-hour observation period, moreover, a daily follow-up period of 10 days by phone call was planned with the owners to evaluate any delayed systemic and local side effects.

**Table 1:** Animal characteristics (sex, age, weight) and group subdivision

**Intravenous Continuous Rate Infusion Group**					
Horse #	Sex	Age (years)	Weight (kg)	CRI duration (minutes)	Total dexmedetomidine dose (µg/kg)
1	Gelding	12	550	103	1.71
2	Gelding	10	560	65	1.08
3	Mare	11	500	105	1.75
4	Gelding	11	580	105	1.75
5	Gelding	7	430	125	2.08
6	Mare	10	512	90	1.50
7	Mare	10	480	108	1.80
8	Mare	14	550	129	2.15
9	Mare	9	550	102	1.70
10	Gelding	13	620	130	2.16
**Subcutaneous Group**					
Horse #	Sex	Age (years)	Weight (kg)	SC administrations (N°)	Total dexmedetomidine dose (µg/kg)
1	Mare	6	523	3	6
2	Stallion	13	550	2	4
3	Mare	6	555	3	6
4	Gelding	18	388	2	4
5	Mare	9	512	3	6
6	Gelding	7	418	2	4
7	Gelding	14	470	2	4
8	Mare	8	500	2	4
9	Mare	9	550	3	6
10	Mare	12	516	3	6

### Anaesthetic protocol description

The full description of the clinical procedures has been reported in detail elsewhere [19].

Briefly, before general anaesthesia for magnetic resonance examination, each horse received IV acepromazine (Prequillan 1%, FATRO S.p.A., Italy) at 0.03 mg/kg then, after 15 min all animals were sedated with 10 µg/kg of IV detomidine (Domosedan, Orion Pharma S.r.l., Italy). Induction of anaesthesia was achieved with IV diazepam (Ziapam, Laboratoire TVM, France) at 0.08 mg/kg followed by IV ketamine (Ketavet 100, MSD Animal Health S.r.l., Italy) at 2.5 mg/kg.

Inhalatory anaesthesia was maintained with isoflurane (Isoflo, Zoetis Italia S.r.l., Italy) adjusted to provide an end-tidal concentration of 1.3%, delivered in a mixture of oxygen (O_2_) and air, so as to maintain the inspired O_2_ fraction (FiO_2_) between 60 and 65% (Datex Ohmeda S5, GE Healthcare, Italy). All horses were mechanically ventilated to maintain an EtCO2 between 35 and 45 mmHg (Mallard 2800 C-P MRI compatible, AB Medical Technologies Inc., USA). Throughout the intra-anaesthetic period, ringer’s lactated solution (Ringer Lattato, S.A.L.F., Italy) was administered IV at 2 mL/kg/h. Moreover, dobutamine (Miozac, Fisiopharma S.r.l., Italy) was administered IV by an infusion pump (Mindray SK-500II, Mindray Medical Italy S.r.l., Italy) to maintain a mean arterial blood pressure ≥ 70 mmHg.

### Blood sample collection

Dexmedetomidine was administered at 15 min from induction, after patient preparation for the magnetic resonance examination and anaesthesia monitoring. Specifically, horses in the CRI group received DEX 1 µg/kg/h IV CRI, diluted at 20 µg/mL in saline solution (NaCl 0.9%, B Braun, Germany) delivered by a syringe pump (Mindray SK-500II, Mindray Medical Italy S.r.l., Italy) up to the end of the general anaesthesia. Animals in the SC group received at the level of the neck a subcutaneous injection of DEX at 2 µg/kg using a 2.5 mL syringe with a 22-gauge × 32 mm needle (RAYS, SPA, Italy) that was repeated every 60 min up to the end of the diagnostic procedure. Both the CRI duration and the number of SC administrations were influenced by the duration of the diagnostic procedures.

Blood samples (2.5 mL each) were collected through a 20-gauge × 51 mm catheter (Surflo, Terumo Europe N.V., Belgium) placed in the metatarsal artery before DEX administration (time 0) and during IV CRI administration at 5, 10, 15, and thereafter at 10-minutes intervals up to the end of the diagnostic procedure; and after repeated SC administration at 10-minutes intervals up to the end of the diagnostic procedure. During the recovery phase after general anaesthesia, blood sampling was scheduled at 10, 30, 60, 90, 120, 180, and 240 min after IV CRI discontinuation; and at 90, 120, 180, 240, 360, 480, 720, and 1440 min after the last SC administration. Since the arterial catheter was removed at the end of the diagnostic procedure, blood samples during the recovery phase were collected by a venous catheter inserted in the controlateral jugular vein only for this purpose to avoid any possible cross contamination by DEX administered during the CRI or any other administered drug.

Considering the particular characteristics of equine patients during the recovery phase, the actual number of samples collected during this period was influenced by the possibility for the operator to approach each animal.

Samples were immediately transferred into tubes containing a clot activator and centrifuged to collect serum, then samples were stored at − 80 °C for maximum 1 month (based on previous stability testing) until the DEX concentrations were measured.

### Dexmedetomidine analysis and validation

Dexmedetomidine was extracted from serum samples according to the method described by Di Cesare et al. (2019) with slight modifications [28]. Briefly, equine serum samples (500 µL) were treated with 50 µL of an internal standard solution (4.5 µg/mL tolazoline hydrochloride in methanol), and then 5 ml of acetonitrile were added to allow protein precipitation. The mixture was vortexed for 10 min and then centrifuged at 3000 *g* for 10 min. The rest of the extraction procedure and mass spectrometry analysis by LC-MS/MS were carried out according to the method described by Cagnardi et al. (2017) [29] (see Additional file 1). The intra-laboratory validation was performed in compliance with the recommendations defined by the European Community [30] and with international guidelines [31].

Validation data of DEX are reported in Table 2. The calibration curves were prepared in equine blank serum with six spiked solutions obtained by diluting the original stock solution of DEX hydrochloride (1 mg/mL + internal standard, 4.5 µg/mL tolazoline hydrochloride) to achieve concentrations ranging from 0.1 to 20 ng/mL. Dexmedetomidine (> 99% pure) was purchased from Tocris (Milan, Italy), and tolazoline (> 99% pure) was purchased from Sigma-Aldrich (Milan, Italy) and used as internal standard for DEX quantification. All salts and solvents were of LC-MS analytical grade (Sigma-Aldrich, Milan, Italy; or Carlo Erba Reagenti, Milan, Italy). There was a linear relationship (r^2^ > 0.996) between the drug concentrations and the area of the peak over the investigated range. The intraday repeatability was measured as a coefficient of variation (%) from six replicates of three concentrations, whereas trueness (%) was measured as the closeness to the concentration added on the same replicates. The results fell within the accepted ranges for precision and trueness (Table 2). A limit of quantification (LOQ) value of 0.019 ng/mL and a limit of detection (LOD) value of 0.014 ng/mL were observed, respectively. The specificity of the method was demonstrated by the absence of interference in 20 blank equine serum samples at the DEX retention time.

**Table 2:** Intra-laboratory validation parameters of analytical method for dexmedetomidine in equine serum samples

Parameter (units)	Dexmedetomidine
LOQ (ng/mL)	0.019
LOD (ng/mL)	0.014
Trueness (%)	99.1–101.2
Intra-day repeatability (CV%)	6.2–11.1
Recovery (%)	99.3 ± 4.1

*LOQ *Limit of quantification, *LOD *Limit of detection, *CV *Coefficient of variation; Trueness and Intra-day repeatability are reported as range values; Recovery is reported as mean ± SD (*n* = 20);

### Pharmacokinetic analysis

Pharmacokinetic parameters were determined from serum concentration–time experimental data using the Phoenix WinNonLin V.8.3 software (Pharsight Corporation, USA), which allows compartmental and non-compartmental analyses. All data points were weighted by the inverse square of the fitted value [32].

Dexmedetomidine disposition following either IV CRI or repeated SC administration in horses was described by standard non-compartmental analysis. The peak concentrations, C_max_, and the time to peak, T_max_, were obtained by visual inspection from the experimentally observed data. The elimination half-life (t_1/2_λ_z_) was calculated as ln_2_/λ_z_. The area under the concentration-time curve from administration to the last measurable concentration (AUC_0-last_) and the area under the first moment curve (AUMC_0-last_) were calculated using the trapezoidal method. Concentrations of DEX resulting below the LOQ were removed from the pharmacokinetic parameters calculation.

In the analyses of repeated administration, it has been assumed that DEX followed linear dosing, as the majority of drugs and as previously stated for repeated extravascular administrations of the same drug in horses [12]. The mean residence time (MRT_0-last_) was determined after SC administration, by using the following equation: MRT_0-last_=AUMC_0-last_/AUC_0-last_ whereas for CRI administration it was adjusted by infusion time as follows MRT_0-last_= (AUMC_0-last_/AUC_0-last_) – (T_inf_/2), where T_inf_ is the length of infusion.

### Statistical analysis

Sample size calculation indicated that a minimum of 20 patients (*n*°=10 per group) were sufficient to obtain power values > 80% with an effect size of 0.8 (high) at an alpha level of 0.05. The distribution normality of the determined pharmacokinetic parameters was assessed with the Kolmogorov-Smirnov test. The principal kinetic parameters of DEX were compared after IV CRI and repeated SC administration using unpaired t-tests with Welch’s corrections (variances unequal) (InStat 3.0, GraphPad Software). Differences with *p* < 0.05 were considered significant.

## Results

All horses successfully completed the study and regardless of group, none of the animals enrolled showed any signs of systemic adverse reactions. Moreover, for the animals in the SC group, no reactions at the site of administration occurred. Finally, none of the animals included in the study experienced prolonged hypotension. In all patients of both groups, the dobutamine rate ranged between 0.2 and 0.8 µg/kg/min.

Twenty horses of different breeds were included in the study: five geldings and five mares received DEX as intravenous CRI; one stallion, three geldings and six mares received DEX as repeated SC administration. Mean age resulted in 10.7 ± 2.0 and 10.2 ± 3.9 years for group CRI and SC, respectively, whereas the mean weight was 533 ± 54 kg in the former and 498 ± 57 kg in the latter group with no significant differences between them.

In the CRI group, the mean duration of IV CRI was 106 ± 20 min (minimum 65; maximum 130). In the SC group, 5 out of 10 horses received two subcutaneous administrations and 5 out of 10 horses received three administrations.

**Fig. 1:**
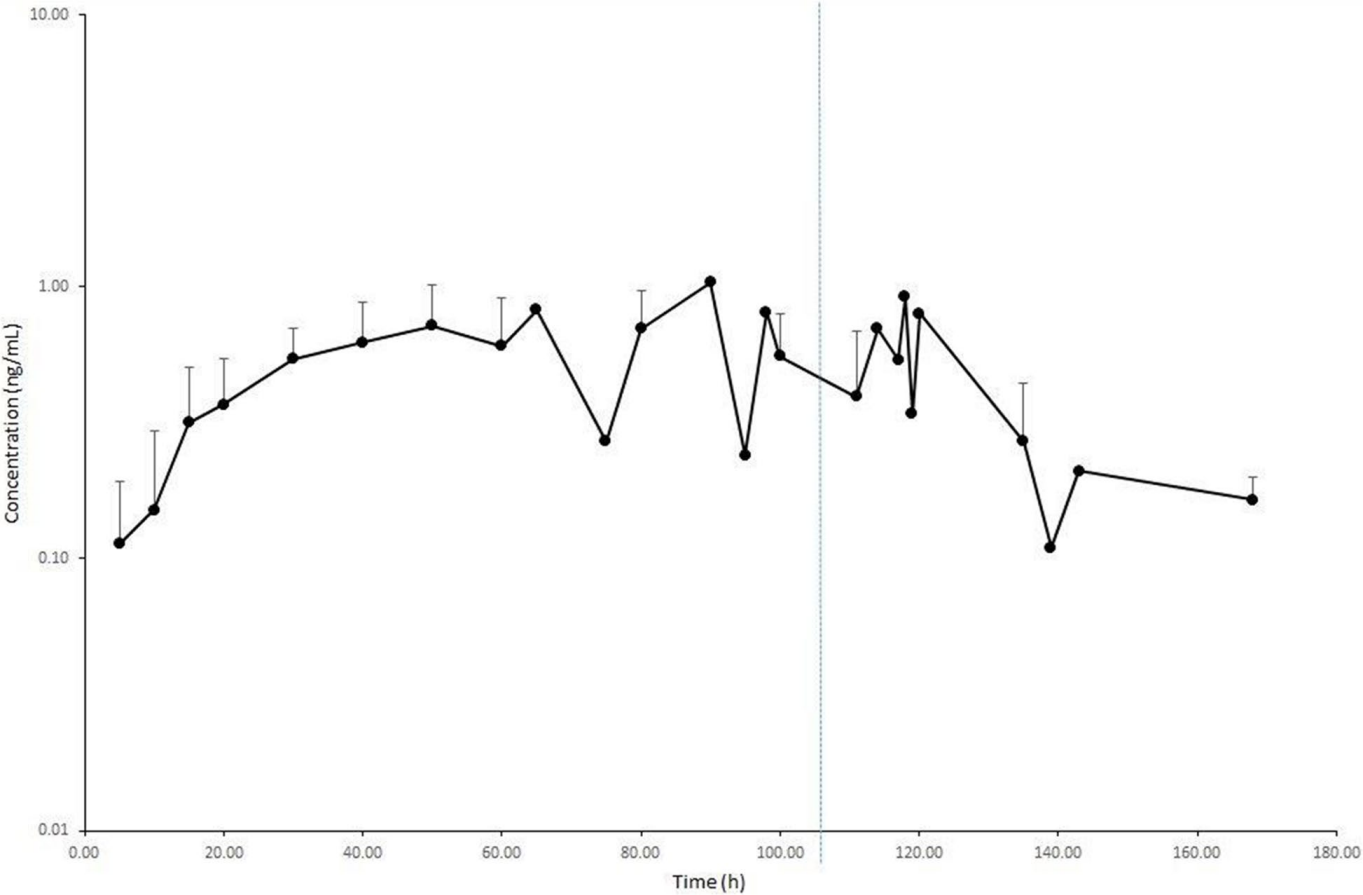
Dexmedetomidine (DEX) serum concentration + SD following intravenous continuous rate infusion (IV CRI) at 1 µg/kg/h in anaesthetized horses (*n* = 10). The blue dashed line indicates the mean time of IV CRI discontinuation

**Fig. 2:**
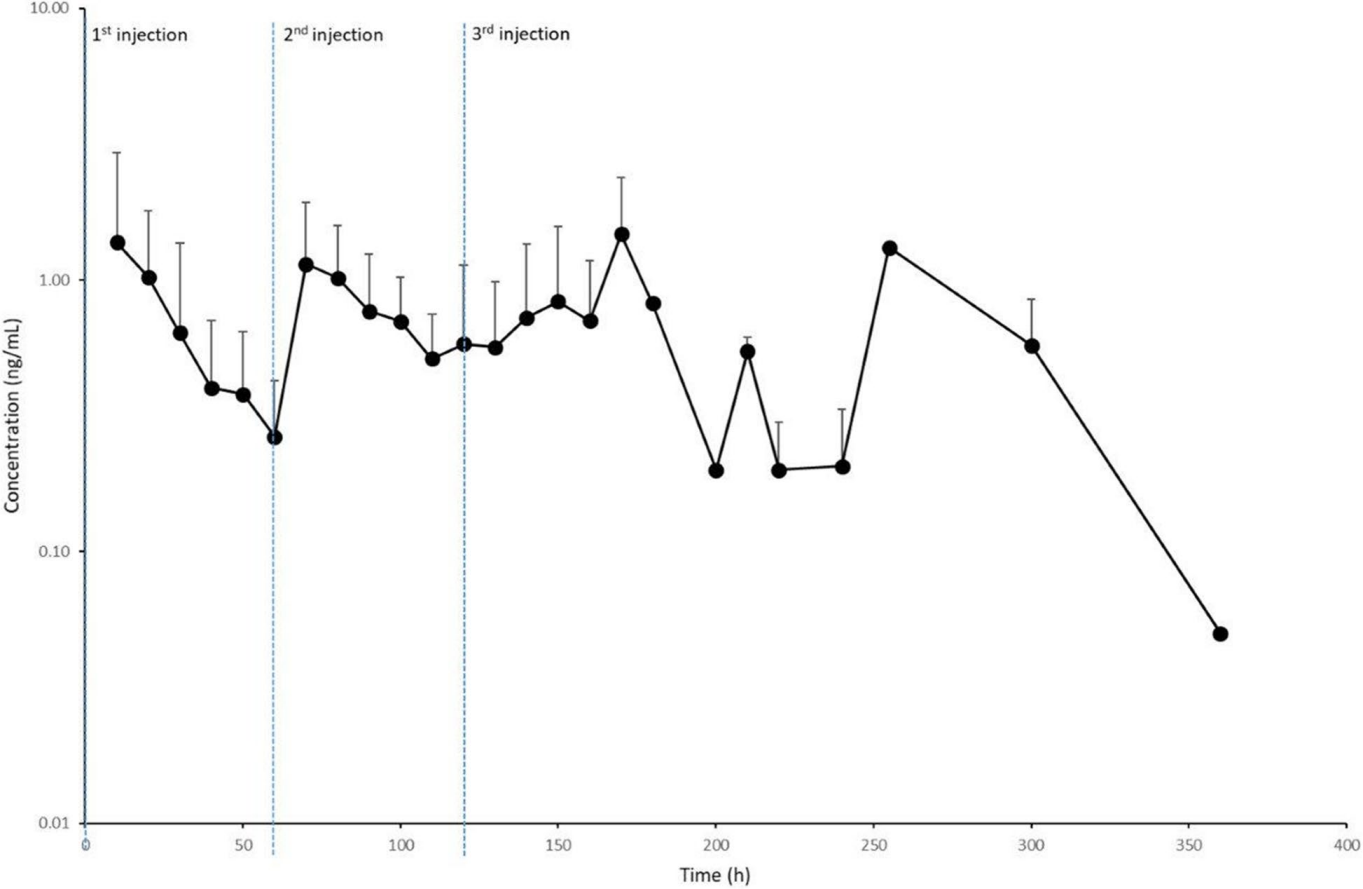
Dexmedetomidine (DEX) serum concentration + SD following repeated subcutaneous (SC) administration at 2 µg/kg in anaesthetized horses (*n* = 10). The blue dashed lines indicate the repetition of SC doses that occurred at 60 and 120 min

Pharmacokinetic parameters data resulted normally distributed and are reported in Table 3 as mean ± standard deviation, whereas the concentration-time curves for DEX concentration in equine serum are illustrated in Figs. 1 and 2 for CRI and SC groups, respectively. The elimination half-life was calculated in 7 out of 10 animals in the CRI group, as after CRI discontinuation serum concentrations rapidly reached the LOQ.

**Table 3:** Mean and standard deviation of pharmacokinetic parameters calculated with non-compartmental analysis after intravenous continuous rate infusion (CRI; 1 µg/kg/h) or repeated subcutaneous (SC; 2 µg/kg) administration of dexmedetomidine in twenty horses

Parameter	Unit	CRI group (*n* = 10)	SC (*n* = 10)	*p*-value
C_max_	ng/mL	0.83 ± 0.27	1.14 ± 0.71	0.22
C_last_	ng/mL	0.29 ± 0.21	0.24 ± 0.22	0.59
T_max_	min	57.0 ± 13.4	105.5 ± 29.9^a^	0.0002
T_last_	min	120.5 ± 28.0	230.5 ± 68.2^a^	0.0002
t _½ λz_	min	18.0 ± 10.0	94.8 ± 69.8^a^	0.0075
AUC_0-last_	min^a^ng/mL	63.7 ± 29.3	83.2 ± 60.5	0.37
V_dz_	mL/kg	390.09 ± 204.21	-	-
Cl	mL/min/kg	16.26 ± 8.07	-	-
AUMC_0-last_	min^a^min^a^ng/mL	4371.9 ± 2526.2	5197.8 ± 5381.9	0.66
MRT_0-last_	min	11.7 ± 6.2	55.8 ± 19.7^a^	0.0001

C_max_ = maximum concentration observed; C_last_ = last concentration observed; T_max_ = observed time for C_max_; T_last_ = observed time for C_last_; t_½ λz_ = elimination half-time; AUC_0–last_ = area under serum concentration–time curve from 0 to last concentration; V_dz_ = volume of distribution based on the terminal phase; Cl = body clearance; AUMC_0–last_ = area under moment curve from 0 to last concentration; MRT_0–last_ = mean residence time from 0 to last concentration. ^a^Significantly different (*p* < 0.05).

## Discussion

Dexmedetomidine is well-recognized as a useful and beneficial adjuvant within balanced protocols for sedation and anaesthesia in equine patients [6].

As a part of a larger clinical study [19], the authors in the present work want to increase the current knowledge on DEX pharmacokinetics in horses, by defining and comparing the pharmacokinetic profile of this drug following IV CRI and repeated SC administration. In particular, this is the first study that reports the pharmacokinetics of DEX administered subcutaneously.

Amongst the different administration routes, the SC injection demonstrated variability in plasma/serum drug concentrations between subjects that sometimes can result in clinical fluctuating efficacy [24, 25]. Besides drug concentration variability, which can be considered a drawback, the SC route presents specific characteristics which have led other authors to speculate on its usefulness in clinical applications [24]. In both humans and animals, the SC administration is performed within the fatty layer of the SC tissue just underneath the skin [23, 27]. Due to the minor vascularization of the SC compartment, the drugs demonstrated to diffuse very slowly and at a sustained rate of absorption, being able to mimic the pharmacokinetic behaviour of a CRI [24, 27].

Considering the absence of data regarding SC administration of DEX in veterinary medicine, the authors selected the dose based on a preliminary investigation. Briefly, a dose of 1 µg/kg (at 60-minutes interval) based on human literature [26, 27] was evaluated but it failed to produce a stable anaesthetic plane comparable to a 1 µg/kg/h CRI (unpublished data). For this reason, the dose of DEX administered as repeated SC injection was increased at 2 µg/kg maintaining the 60-minutes interval.

Conversely to other studies [18, 33], the authors did not administer an initial bolus of DEX by IV route before CRI initiation. The bolus might have influenced the pharmacokinetic behaviour of DEX administered by CRI and might have complicated the comparison of the two routes, therefore the authors preferred the administration of detomidine during the premedication phase to rapidly achieve a steady-state clinical condition. In fact, the use of different alpha-2-adrenergic receptor agonists for premedication and CRI during general anaesthesia is an approach already described by other authors to promote balanced anaesthesia [3, 34].

As reported by other authors, DEX pharmacokinetics is affected by many factors and it is not simple and properly useful to compare results deriving from different clinical settings and designed for different clinical purposes [10, 12, 13]. Indeed, when considering animals undergoing general anaesthesia, the main factors able to influence DEX pharmacokinetics are represented by the co-administration of drugs within the balanced protocol and the hemodynamic effects on the cardiovascular system induced by anaesthesia itself [6]. Nevertheless, the pharmacokinetic parameters recorded in this study are generally in line with those reported by other authors that describe DEX pharmacokinetics in conscious and anaesthetized horses [10, 12, 15–18]. In fact, following CRI administration, DEX demonstrated a short elimination half-life (t _½ λz_ = 18.0 ± 10.0 min), a large volume of distribution (V_dz_ = 390.09 ± 204.21 mL/kg), a rapid clearance (Cl = 16.26 ± 8.07 mL/min/kg), and a short mean residence time (MRT_0-last_ = 11.7 ± 6.2 min) (Table 3). When comparing the Cl value with those reported in other studies [12, 15–17], it resulted more rapid. This finding could be still explained by the different study settings, where the hemodynamic alterations induced within the balanced anaesthetic protocol by the pharmacological modulation of cardiac output could have contributed to lower the Cl value compared to conscious and sedated horses [12, 15–17]. Moreover, the correlation of Cl to equine cardiac output as reported by Toutain and Bousquet-Melou (2004) [35], for healthy and non-anasthetised horses showed a maximum Cl value of 27.5 mL/kg/min for a drug eliminated by a first pass effect by both liver and kidney, since it is equal to about half of the cardiac output. In our horses undergoing general anaesthesia in a balanced protocol, the Cl resulted much lower than this reference value and it represents approximately 30% of cardiac output, hence suggesting that the impact of anaesthetic protocol on the cardiac output could have a relevant influence on this parameter.

Following repeated SC administration, the apparent t _½ λz_ (94.8 ± 69.8 min) and the MRT_0-last_ (55.8 ± 19.7 min) resulted higher than by CRI, however, this finding could be explained by the presence of a flip-flop phenomenon induced by a rate of absorption slower than the rate of elimination by this route of administration.

In this study, maximum concentration (C_max_) and last concentration (C_last_) did not differ significantly between the two routes of administration. Regarding the former, the main explanation could be probably related to the high inter-subject variability in horses of the SC group, a common result already previously reported for this route of administration [23]. Moreover, this aspect could be further influenced by the different number of administered doses that were correlated with the length of the diagnostic procedure. For C_last_, it must be pointed out that this parameter is strictly related to the time of the last concentration (T_last_) that differed significantly between groups thus, even though no statistical significance was reported, it is difficult to state that C_last_ in the two groups (0.29 ± 0.21 and 0.24 ± 0.22 ng/mL for IV CRI and SC group, respectively) was comparable.

Time-to-maximum concentration (T_max_ 105.5 ± 29.9 min) and T_last_ (230.5 ± 68.2 min) resulted higher in the SC group; regarding T_max_, this parameter was probably affected by the different number of adimistered doses in the SC group. The highly different T_last_ (by IV CRI 120.5 ± 28.0 min) could be explained by the fact that, after CRI discontinuation, DEX serum-concentration data resulted frequently below the LOQ, as also supported by the rapid elimination half-life and clearance.

As mentioned above for C_max_, also the area under the curve from 0 to the last concentration (AUC_0-last_) and the area under the moment curve (AUMC_0-last_) were comparable between groups mainly due to the high interindividual variability and the different dosages employed in the SC group. Considering the interindividual variability in the number of SC administrations or length of CRI and in blood sampling, it was not possible to calculate the bioavailability in the 24 h, but the tentative calculation of the bioavailability (adjusted by dose) in the first 60 min, the time interval with a comparable number of sampling point and dosing, resulted of 63% showing a quite good absoprtion of the SC administration.

In general, considering the range of DEX effective doses reported in the literature for equine patients, is possible to state that the DEX serum concentrations reached in this study have to be considered effective regardless of the treatment group [10, 12, 13, 15–18].

As stated by the same authors in the clinical study [19], the two administration routes of DEX within the intranaesthetic period demonstrated comparable effects on the physiological parameters and the SC route proved to be better in improving the quality of recovery from anaesthesia. Considering the results of this study, the present pharmacokinetic investigation strongly supports that statement, so as the hypothesis that a regimen based on repeated SC administration of DEX at the dose of 2 µg/kg at 60-minutes intervals could have a comparable pharmacokinetic profile of a CRI at the dose of 1 µg/kg/h and to provide the equine patient with a safer and high-quality recovery from general anaesthesia.

This study presents some limitations, all related to the execution of the study in a clinical setting that avoided the application of a classical standardized cross-over design and that contributed to increasing the variability of PK parameters. The number of SC administrations and the CRI dose were influenced by the diagnostic procedure duration defined based on the clinical requirements of the patients, leading to a further increase in the variability of PK results. Moreover, pharmacokinetic parameters might have been additionally influenced by the simultaneous administration of the other anaesthetic drugs used in the premedication and induction phase of the balanced anaesthetic protocol, so as by the anaesthesia-related variation of the physiological parameters. Finally, since the recovery of horses was unassisted, it was not always possible to approach the patient for blood sampling collection at all the scheduled time points, with a consequent reduction in the number of serum DEX quantifications during the elimination phase of the drug. Further studies are advocated to evaluate and compare the pharmacokinetic profile of DEX administered subcutaneously in different clinical settings, trying to include this alternative delivery route within a balanced protocol for equine sedation.

## Conclusions

In conclusion, in horses undergoing general anaesthesia for magnetic resonance examination a regimen based on DEX repeated SC administration at the dose of 2 µg kg^−1^ at 60-minutes intervals showed during the intranaesthetic period a pharmacokinetic profile comparable to a CRI at the dose of 1 µg kg hour^−1^. Moreover, the absence of any systemic or local adverse reaction together with the beneficial pharmacokinetic behaviour of the SC administration within the recovery phase, suggests and supports DEX administration by this alternative route within a balanced anaesthetic protocol for equine patients undergoing general anaesthesia for diagnostic procedures.

### Supplementary Information

**Additional file 1: LC-MS/MS conditions description and Supplementary table S1 (DOC). **Instrumental method Liquid Chromatography tandem Mass Spectrometry conditions for dexmedetomidine quantification in equine serum.

## Data Availability

The datasets used and/or analyzed during the current study supporting our results are included in the article. The data are available from the corresponding author upon reasonable request.
