# Supplementary material for: Pharmacokinetics of dexmedetomidine in anaesthetized horses following repeated subcutaneous administration and intravenous constant rate infusion
Source: BMC Vet Res. 2023 Dec 9;19:264. doi: 10.1186/s12917-023-03831-w (PMC10709979; doi:10.1186/s12917-023-03831-w)
Supplement: Supplementary file 1 — Additional file 1: LC-MS/MS conditions description and Supplementary table S1 (DOC). Instrumental method Liquid Chromatography tandem Mass Spectrometry conditions for dexmedetomidine quantification in equine serum. [file 12917_2023_3831_MOESM1_ESM.docx]

**Instrumental method Liquid Chromatography tandem Mass Spectrometry conditions for dexmedetomidine quantification in equine serum**

Mass spectrometric analysis was performed using a LTQ XL ion trap (Thermo Fisher Scientific, San Jose, CA, USA) equipped with a heated electrospray ionisation probe operating in the positive ion mode under the following conditions: sheath and auxiliary gas flow: 25 and 5 arbitrary units, respectively; ion spray voltage: 3.5 kV; capillary temperature: 300° C; capillary voltage: 29 V; and tube lens: 70 V. The collision energies that were determined to be necessary for fragmentation in MS^2^ of the molecules of interest, precursor ions, product ions and collision energies are shown in the Supplementary table. S1 The Xcalibur (version 2.1) data acquisition software from Thermo Fisher Scientific was used (Thermo Fisher Scientific, San Jose, CA, USA).

Supplementary table S1. Instrument acquisition data for the analysis of dexmedetomidine and the internal standard (IS) by Liquid Chromatography tandem Mass Spectrometry.

| **Compound name** | **Precursor ion**  **[M-H]^+^**  **(m/z)** | **Collision energy**  **(%)** | **Product ion**  **MS^2^**  **(m/z)** |
| --- | --- | --- | --- |
| Dexmedetomidine | 201 | 26 | 95 |
| Tolazoline (Internal Standard) | 161 | 45 | 77 |
